# A Fatal Case of Paraplegia, from an Uncommon Cause; Together with an Account of the Dissection, and Some Observations Thereon

**Published:** 1823-05

**Authors:** Edward Harrison


Art. VII.—*A fatal Case of Paraplegia, from an uncommon Cause; together with an Account of the Dissection, and some Observations thereon.* Communicated by Edward Harrison, M.D.

Miss R. S. aged eleven years, of a fine complexion, a very delicate form, and in feeble health, complains of great weakness and emaciation. These symptoms have rapidly increased of late. She has scarcely the power of walking or standing alone. Appetite is small, and uncertain. Her bowels are generally constipated. The motions are black, and emit a sulphureous smell. She is frequently called upon to make water, and always suddenly. The disorder was first distinguished by pains in the small of the back, in the lower region of the abdomen, about the hips, the fore-part of the thighs, and legs. She says, that she cannot bear to extend her lower limbs, when in bed, from the pain which the effort produces.

The disorder commenced more than three years ago. She then resided upon the sea-coast; and, till within the last few days, has been under the care of a fashionable physician and an experienced surgeon, who gave it as their opinion that she was suffering from inflammation in the hip-joints.

On examination, I found the twelve dorsal vertebræ rather protuberant; the three upper lumbar vertebræ were also too much raised, especially the middle one: together they formed a small arch. Most of the ribs were a little displaced in front, and projected irregularly forward. The breast was peaked, and unusually narrow. Considering the patient to labour under a decided spinal complaint, a firm bandage was put round her chest and loins; she was then laid horizontally upon a flat mattress,—a position which she could not be induced to give up during the remainder of her illness.

*April* 17*th*, 1821.—Miss R. S. has had a good night, and feels easier this morning, in consequence, as she believes, of having adopted the recumbent posture, and the support derived from her bandage. There has been one dark-coloured motion. She has seldom made water, and it is voided with greater freedom. Her legs and feet, which had for a long time past been deadly cold, were this evening warm and moist. She is observed either to keep picking her nose without a moment's cessation, or to thrust and turn her fingers in one nostril or the other, with force enough to make them raw, and frequently discharge a few drops of blood.

18*th*.—She had two motions yesterday, and five during the night, from having taken some aperient pills. The fæces have a more natural appearance, and her urine continues to be discharged with ease. Appetite is good, and digestion so much improved, that she is less restricted in diet. The lower limbs are warm, and free from pain, except at the top and inside of her thighs, where they are tender, stiff, and contracted.

28*th*.—Debility and emaciation continue to increase. Feverish paroxysms, commencing with slight shiverings, recur daily, at uncertain intervals. They are accompanied with a hot and dry skin, but are seldom followed by any perspiration. Urine is high coloured, and deposits a lateritious sediment. The bowels incline towards costiveness, and the fæces have a natural appearance. Although the pains in the abdomen and thighs have gradually subsided, the febrile symptoms are rather aggravated. The patient, for the last few days, has had a teazing cough, which is imputed to her having caught cold. It is accompanied with increase of fever, and with sharp, darting pains in different parts of the chest. The physician above referred to, being consulted for this new attack, delivered it as his opinion that she was probably now suffering from phthisis pulmonalis.

*June* 5*th*.—Though the catarrhal symptoms and pains have entirely left her, the fever still returns, and is even more violent. The weakness and extenuation have also advanced so much within the last few days, as to occasion, in the minds of her friends, the most alarming apprehensions. Under the influence of this impression, another popular physician was applied to, who, having carefully inquired into all the circumstances, was led to imagine the seat of the disorder to be in the mesenteric glands.*

12*th*.—From the date of the last report, the little sufferer had regular attacks of hectic fever, which invaded her generally in the evening. The appetite was nearly lost; and she grew visibly weaker till June 21st, when she expired, about noon. The ir-

* See Dr. Jebb on *Paralysis, &c.* Case ix.

ritation and tenderness of the nostrils suffered no diminution during the whole period of her illness.

The examination was performed early on the following morning, by a physician who has paid great attention to morbid anatomy, the family surgeon, another medical gentleman, and myself.

The whole person appeared extremely thin, except that the abdomen was somewhat swollen. The skin covering it was a little discoloured, and had a gangrenous hue. Upon removing the abdominal coverings, the intestines were discovered to be healthy, and slightly distended with inclosed air.

On opening the chest, the lungs had collapsed. The pleura pulmonalis et costalis was studded with small hard substances, bearing a resemblance to grains of sand. Water was detected on both sides the folds of the pleuræ, and also in the pericardium. When cut, the lungs every where exposed an innumerable quantity of small tubercles, dispersed through their substance in every direction. None of them had proceeded to suppuration. At the upper and inside of the chest, immediately under the pleura, on the right side, two vomicæ, each about the size of a pigeon's egg, were discovered, containing very thick curdy pus. The heart was of its proper size, and healthy.

In the abdomen, the omentum and peritoneal covering of the intestines were in a sound state. The former adhered to the uterus and bladder. A pint, or more, of a thin, pale, whey-coloured fluid was discovered in the pelvis.

The liver was greatly enlarged. It extended to the ribs on the left side, and almost surrounded the spleen. It descended nearly to the top of the os ilium, on the right side. Its peritoneal covering showed the same granulated appearance as the lungs, but less in quantity. The gall-bladder was very small, and contained a little yellow bile.

The peritoneum over the spleen discovered the same sandy characters, and displayed in some parts a false membrane lying upon it. The interior of the spleen manifested the same tuberculated substances as the lungs. Both kidneys had enlarged, and in particular the left.

A few mesenteric glands were somewhat tumid, especially about the head of the colon. None of them manifested any disposition to suppurate.

In the loins, on each side, we found a large psoas abscess, contained in a bag of unusual thickness. The two had a communication with each other across the spine, between the first and second superior lumbar vertebræ. That on the right extended from the ninth dorsal vertebra to a little below Poupart's ligament; it enclosed a pint of thick pus. On the left, the abscess contained a quart of purulent matter. It commenced also at the ninth, or same dorsal vertebra, and ran on the top of the spine as far as the third lumbar vertebra; from thence it passed below Poupart's ligament to the middle of the inside of the thigh. The anterior crural nerves, on each side, ran through the abscesses. Their outer coats had a chalky appearance, probably from having been so long immersed in the purulent fluid; they seemed to be otherwise free from disease. The three lower dorsal vertebræ were laid bare in front, by the abscess running upon them. Between the first and second lumbar vertebræ, the cartilaginous substance was wholly destroyed, and the vacant part filled with purulent matter, similar to what the abscesses contained. The spinal marrow in the part, being exposed to view, was unusually soft. On raising these two bones, a complete separation was found between them: they were quite loose, and showed no signs of union, or of any attempt at anchylosis having taken place. A portion of the upper surface was superficially destroyed in both of them. Neither these nor any of the other vertebræ showed the slightest mark of disease; they manifested their natural hardness and firmness. The intervertebral substance, except the portion of it which was placed between the first and second lumbar vertebræ, was also perfectly sound, and preserved its usual colour. All the viscera were very pale, and the vessels were nearly empty of blood.

On laying open the hip-joints, we found them, and their capsular membranes, in an healthy condition; nor did they display the slightest trace of having suffered from any former disease.

In order more clearly to comprehend some of the distressing symptoms of this obscure complaint, it may be useful to premise a short description of the *great sympathetic nerve*, and its anatomical connexions. This extraordinary production has two terminations; one of them is situated within the skull, from which it proceeds along the neck, the chest, and abdomen, to the bottom of the back, where it gradually disappears. In this long course it is disposed near to the spine, and sends branches to be distributed upon the viscera of the chest, abdomen, and pelvis. In the neck, and within the skull, it is connected to the several nerves which are expanded on the face.

It has been already observed, in speaking of the dissection, that the psoas abscesses began on both sides of the spine, at the ninth dorsal vertebra, and from thence extended along the thighs to some distance below Poupart's ligaments.

The anterior crural nerves, in their course from the spine, ran nearly through the centre of these abscesses, in a longitudinal direction, emerging from thence in the usual way; they proceeded along the fore-part of the thighs and legs, to the bottom of the feet, distributing their fibrils to the neighbouring muscles. The patient's inability to walk, and almost to stand, were (as I conceive) produced chiefly by the diminished energy of these nerves. Another portion of the lumbar division passed through the same purulent collection, in its way to the bladder and great intestines. The operations of these viscera, it is well known, are chiefly regulated by nerves issuing out of the lumbar plexuses: as these are supplied from the spine, it follows that the functions dependent upon them must be materially influenced by the condition of the lumbar vertebræ. In the present instance there was difficulty in discharging the urine; on other similar occasions I have known an ammoniacal odour emitted. It is well ascertained that the excrementitious matter does not obtain its proper consistence, or peculiar fetor, until its arrival at the head of the colon: here, and probably likewise in the arch of the colon, the figure of the stools, and their fecal smell, are further evolved. According to this explanation, which will be more fully detailed in my Essay on the Pathology and Diseases of the Spinal Nerves, the difficulty in making water,—the dark complexion and sulphureous smell of the excrement,—admit of a satisfactory illustration, on the principle already laid down.

The remaining portions of the lumbar division must also have suffered, from the same cause, in their way from the lumbar vertebræ to their final destination. The severe sufferings of this delicate patient about the hips, the thighs, and in the lower part of the back and abdomen, arose from the disordered condition of the lumbar nerves ramifying upon these several regions of the body, and not, as was erroneously asserted, from any malady in the hip-joint.

The uneasy sensation, so much complained of, in the nostrils, was, I conceive, induced by the irritation of the psoas abscesses, communicated through the great sympathetic to a branch of the fifth pair of nerves, which is distributed upon the nostrils. A similar effect, and operating (as it would seem) through the same medium, has been frequently elicited by the lodgment of worms in the intestinal tube.

Should objections be raised against the principle laid down to explain the phenomena, on account of the great variety of symptoms displayed in this case,—their apparent want of connexion with each other,—and their bursting forth, simultaneously, in so many different parts of the body,—we may remark, that manifestations more complicated have been frequently observed, on other occasions, from the disorder of a single nerve.

Such, it is obvious, must be the result when the offending cause is applied to the sheath of a large bundle of nerves; because, as the noxious effects are usually perceived only at their ultimate ramifications, these must be revealed in the various places and tissues upon which the fibrils are distributed.

The anatomical examination of this delicate girl was confined to the chest and abdomen. Of the different viscera seated in them, scarcely one was wholly sound at the time of death. Had this fatal complaint been subdued, and life been extended only a few years longer, these diseased structures would have kept increasing till some other mortal disease had supervened. The lungs were already deeply tainted, and a few mesenteric glands were also a little swollen. Still nothing had yet arisen, in either of these organs, to sanction the opinions entertained by the very respectable physicians above referred to. In the lungs, two small vomicæ had formed; but not of a magnitude sufficient to excite the hectic, by which the patient was gradually exhausted. The mesenteric taint was much too slight to influence the constitution, or create any present inconvenience. Enough, however, was detected in these and other parts to show that a beautiful flower was doomed to fade, before an opportunity had been given to display its finest bloom.

The psoas abscesses, the true cause of the hectic fever, had never been even suspected while the patient was alive. When she arrived in London, the inflammatory stage having terminated in suppuration, no sharp pain, swelling, tension, or other symptom indicating mischief in the loins or thighs, remained to draw attention to these parts.

In opposing the too-prevalent notions of medical men with respect to pains in the haunches, I am borne out very clearly (as, at least, it appears to me,) by the dissection at present under consideration. On this occasion, two professional gentlemen, deservedly of high character and great experience, were so fully convinced that mischief had actually taken place in the hip-joint, that all the resources of the healing art were exclusively directed, during the period of more than a whole year, with a view to obviate consequences arising from a malady which never had any foundation in nature. Had this protracted disorder been found out in an early stage, its further progress might possibly have been stopped, by suitable remedies judiciously applied.* Even after it proceeded to suppuration, and pus was collected in the thighs, other expedients could have been tried, with a view to cure, or at least to moderate, the severity of the complaint. The true source of the disorder being clearly ascertained at a late period, though it was found to

* Dr. Jebbs's Select Cases of the Paralysis of the lower Extremities.

be incurable, still, in consequence of that knowledge, the amiable sufferer might have been enabled to pass her last moments in greater comfort.

Were this only a solitary example of the kind, I should have felt less inclination to expatiate upon it. Believing, as I do, that some of our most distinguished practitioners are too much inclined to refer directly to the hip-joint every pain and uneasiness which are felt in its immediate vicinity, I wish to caution my brethren not to fall into the common opinion, and surrender their better judgments to the authority of others; but to examine very carefully for themselves, and decide without prejudice.

Of the reflections suggested by a consideration of the foregoing narrative, the ignorance of what was going on, for so many successive months, in the spine, loins, and thighs, is the most striking. A knowledge of the true nature of the complaint in its nascent state, would have led, as already remarked, to the application of suitable remedies, with a fair prospect of subduing the malady, and thereby preventing all the misconceptions into which the attendant faculty were subsequently deceived.

When mistakes are committed in practice, the healing art suffers equally in its usefulness and the estimation of society: it is, therefore, incumbent upon every member of the curative art to exert his best endeavours, however inadequate they may be, to render it as perfect as possible.

In the present instance, all the practitioners confided in were gentlemen of great observation, extensive experience, and in whom the public justly repose the utmost confidence.

The oversight committed on the present occasion shows clearly, as it appears to me, that the phenomena arising from spinal maladies have not hitherto been sufficiently noted. I am, however, of opinion, that the seat and nature of the disorder might, in this case, have been discovered in its early access. After inflammation had given way to the suppurative process, the tensive and darting pains about the back and hips no longer existed to point out the source of the injury. For this reason, observation was no longer directed to the lumbar region. Owing to strong fasciæ in the fore-part of the thighs, the abscesses never produced any circumscribed tumor to indicate their presence; so that, although, on her arrival in London, the patient was viewed naked by a very able surgeon, and made to walk about the room before him, their existence was not suspected. We may therefore conclude, as I have already remarked, either that the symptoms of spinal complaints are very obscure in themselves, or that they have not been carefully recorded. Though affections of the lumbar spine,* and of the

* Bell's Surgery, vol. 5th.

hip-joint, are productive of pains about the haunches, they may generally be distinguished from each other. In the former, there is uneasiness in the small of the back, and generally a deranged condition of the pelvic organs.

Whenever pains are felt in both haunches at the same time, we may conclude that their source is in the spine, because it seldom happens that the two hip-joints are afflicted together. It is quite otherwise with respect to the vertebræ. The nerves issuing from them being directed to each side of the body, whatever disturbs one set acts with equal force upon the other division. Nor should we omit to assist the inquiry by inspecting the parts. When the malady is fixed in the small of the back, pressure upon it excites uneasiness and tenderness; while exercise is accompanied with a sensation of weakness in the loins, and is performed with difficulty.

Disease of the hip-joint is distinguished from the former by the pain which is felt in the acetabulum, on giving a smart rotatory movement to the head of the thigh-bone, or standing with the whole weight on the same limb. In an early stage, the thigh and calf of the leg are reduced in size, as well as the natural fulness and rotundity of the nates. Troublesome pains are usually felt in the knee of the affected side, which, after careful examination, will be found in a healthy condition. It may be still further observed that, in standing, the diseased limb is longer than the other, and the patient does not support his body equally on both legs. The sound one is made to bear more than its due proportion: with the other, he is rather inclined to rest on his toes, than to place the whole weight on the surface of his foot generally. In forming a diagnosis, we must not be misled by the irregularity of the limbs. We have already remarked, that, in hip cases, the affected member is first elongated, and afterwards shortened. Curvatures in the lumbar spine also disturb the arrangement of the pelvis, by altering the relative position of the ossa ilia. In consequence of the situation of the acetabula and heads of the thigh-bones being changed in respect to each other, the same disproportion and want of symmetry is produced in spinal complaints as in those of the hip. The parts surrounding the joint, on pressure, betray some soreness behind the great trochanter. Moreover, the lymphatic glands in the groin are frequently enlarged and tender. We may further remark, that the elongation and wasting of the limb, the pain in the knee, the uneasiness felt in moving the head of the thigh-bone in its socket, are such prominent and characteristic features, that, when they are all present, we may decide, with great confidence, that the patient labours under the hip-disease.

After suppuration had formed, so little inconvenience was perceived in the loins or hips, that attention was no longer directed to these parts. The catarrhal symptoms and shooting pains in the chest, which supervened about this time, seemed to point directly to the lungs as the true source of the consuming hectic. In a few days this important organ resumed its healthy functions, and then the supposed morbid condition of the mesenteric glands became the chief cause of anxiety.

The tender age, the strumous constitution, and pallid aspect of the patient, favoured the latter opinion. This was further confirmed by the uneasiness produced in the small of her back, and darting pains in the belly, whenever she arose from her couch. The want of a chalky or milky appearance, and of mucus or blood, mixed with the fæces, which usually accompany the more advanced stage of this complaint, were admitted to militate against the supposition entertained. After various consultations, the nature and origin of the malady were considered to be very obscure.

The chief part that devolved upon me in this perplexing affair, was to find out the true cause of her sufferings. I was so forcibly struck, at my first visit, with the appearance of her back, that I ventured to declare it as my opinion that she was labouring under a spinal complaint. She was at this time able to walk about in her room, and bear the motion of a carriage. The hectic fever, if any had commenced, was trifling, and escaped notice. Under such circumstances, I did not hesitate to place her in an horizontal situation preparatory to the adoption of ulterior measures for her relief. In the mean time she was seized with catarrh, which preceded such a clearly-marked hectic fever, that I began to entertain so many fears for her recovery that I no longer directed my principal attention to the vertebræ. In order to promote her ease and comfort, she was, as I have already observed, several times prevailed upon to rise from the mattress, and sit up, or go to bed in the usual way. Soon after making the attempt, she experienced such an increase of uneasiness about the hips and lower part of her belly, that she always entreated to be replaced horizontally upon her crib; and could never be induced to leave it for many minutes together, during the remainder of her illness.

1st. My chief object, in publishing this case, is to point out a cause of paraplegia, which has, I believe, been seldom observed in practice. Caries of the vertebræ, accompanied with an injured condition of the interposed substance, is of frequent occurrence; whereas very few examples have, as far as I know, been recorded, in which the complaint derived its origin from, and was entirely confined through its whole course to, the intervertebral substance of a single joint. Neither the vertebræ themselves, nor their involucra, participated in the least: they continued sound, or only suffered externally from the corrosive quality of the pus resting upon them. In all cases where the intervertebral matter is destroyed along with the contiguous bones, the disorder has, I believe, been supposed to originate in the vertebræ, and proceed from them to the intervertebral substance. Here, on the contrary, is an example of paraplegia, in which the vertebræ were unaffected; and it remains to be ascertained, by future enquiries, whether such instances are of frequent or rare occurrence.

2dly. The symptoms of paraplegia have, as I conceive, been less considered and investigated than their real importance deserves. Owing to these circumstances, the disorder has been too often confounded with others, to which it bore little resemblance in nature, and from which it might have been distinguished by paying due regard to the various indications and anatomical structure of the parts implicated. Though the difficulty of distinguishing the early manifestations of this formidable complaint often causes great embarrassment and uncertainty, it is generally marked throughout with such peculiar and characteristic symptoms, that the careful observer may generally discover and distinguish it from all others.

Had the case concluded here, we should have remained ignorant of its nature and origin. The anatomical examination cleared up and removed all the doubts entertained during life. Through it we first discovered that inflammation of the fibro-cartilaginous substance placed between two lumbar vertebræ had proceeded to suppuration. The purulent matter thus formed on both sides of the spine, following the course of the psoas muscles, had actually made its way unobserved below Poupart's ligament, and lodged in the fore-part of the thighs, where it remained concealed under strong fasciæ. If the cause had fortunately been ascertained during the inflammatory stage, the disorder would probably have been arrested in its course, and the suppurative process avoided. In that case, the train of symptoms to which it gave rise would not have formed, and the patient might have been restored to a good state of health.

Having explained in what manner affections of the lumbar spine produce the various symptoms which afflicted the subject of this case, I do not propose at this time to enter into further details: I, however, venture to maintain that, according to my observations, spinal complaints may be known by certain signs and characters, so as not only to enable us to determine their immediate presence, but also to point out from what particular division of the spine they proceed.

This part of the inquiry I am obliged, for want of leisure, to defer to some future opportunity; when I propose to enter fully into the subject, and also to show in what manner distortions of the spine lay the foundation of various nervous disorders.

